# Diagnostic value of repeated comprehensive investigation with CT urography and cystoscopy for recurrent macroscopic haematuria

**DOI:** 10.1002/bco2.300

**Published:** 2023-10-06

**Authors:** Suleiman Abuhasanein, Vanessa Chaves, Ali Moustafa Mohsen, Jasmine Al‐Haddad, Merete Sunila, Viveka Ströck, Tomas Jerlström, Fredrik Liedberg, Jesper Swärd, Truls Gårdmark, Staffan Jahnson, Henrik Kjölhede

**Affiliations:** ^1^ Department of Urology, Institute of Clinical Science, Sahlgrenska Academy University of Gothenburg Göteborg Sweden; ^2^ Department of Surgery, Urology Section NU Hospital Group Uddevalla Sweden; ^3^ Department of Urology Sahlgrenska University Hospital Göteborg Region Västra Götaland Sweden; ^4^ Department of Urology, School of Medical Sciences, Faculty of Medicine, and Health Örebro University Örebro Sweden; ^5^ Department of Urology Skåne University Hospital, Malmö, Sweden and Institution of Translational Medicine Lund University Malmö Sweden; ^6^ Department of Clinical Sciences, Danderyd Hospital Karolinska Institute Stockholm Sweden; ^7^ Department of Clinical and Experimental Medicine, Division of Urology Linköping University Linköping Sweden

**Keywords:** bladder cancer, computed tomography, cystoscopy, haematuria, recurrent, urography, urological neoplasm

## Abstract

**Objectives:**

To perform a descriptive analysis of a series of patients with recurrent macroscopic haematuria after a primary standard evaluation including computed tomography urography (CTU) and cystoscopy negative for urinary bladder cancer (UBC) and upper tract urothelial cancer (UTUC) and to identify potential factors associated with occurrence of recurrent macroscopic haematuria.

**Methods:**

All patients older than 50 years who underwent urological investigation for macroscopic haematuria with both cystoscopy and CTU 2015–2017 were retrospectively reviewed. A descriptive analysis of the primary and later investigations for recurrent macroscopic haematuria was performed. To investigate the association between explanatory variables and the occurrence of recurrent macroscopic haematuria, a Poisson regression analysis was performed.

**Results:**

A total of 1395 eligible individuals with primary standard investigation negative for UBC and UTUC were included. During a median follow‐up of 6.2 (IQR 5.3–7) years, 248 (18%) patients had recurrent macroscopic haematuria, of whom six patients were diagnosed with UBC, two with prostate cancer, one with renal cell carcinoma and one had a suspected UTUC at the repeated investigation. Within 3 years, 148 patients (11%) experienced recurrent macroscopic haematuria, of whom two patients were diagnosed with low‐grade UBC (TaG1–2), one with T2G3 UBC and one with low‐risk prostate cancer. The presence of an indwelling catheter, use of antithrombotic medication, pathological findings at CTU or cystoscopy or history of pelvic radiotherapy were all statistically significant independent predictors for increased risk for recurrent macroscopic haematuria.

**Conclusion:**

In the case of recurrent macroscopic haematuria within 3 years of primary standard evaluation for urinary tract cancer, there was a low risk of later urological malignancies in patients initially negative for UBC and UTUC. Therefore, waiting 3 years before conducting another complete investigation in cases of recurrent macroscopic haematuria might be appropriate.

## 1 INTRODUCTION

Macroscopic haematuria is a common urological condition affecting 0.2%–16% of the population^1^ and may occur because of various underlying oncological or benign conditions.^2^, ^3^ Meta‐analysis of 229 701 patients with macroscopic haematuria showed that the pooled incidence rate for urinary bladder cancer (UBC) is 14%–20%.^4^ Other urological malignancies may occur, for example, renal cell carcinoma (RCC) (2%) and upper tract urothelial cancer (UTUC) (0.75%).^4^ Macroscopic haematuria is considered a serious symptom that requires prompt medical attention and an immediate referral to a urological unit for standard investigation with cystoscopy and computed tomography urography (CTU).^5^ In Sweden, a standardized care pathway (SCP) for patients with macroscopic haematuria in patients aged 40 years or older (the age limit was changed to 50 years or older from 2018) was implemented in 2016 on a nationwide basis to provide a uniform and standardized investigation including a prompt referral, with cystoscopy and CTU done within 1 week.^6^

Despite standard investigations after macroscopic haematuria, a considerable proportion of patients (20% to 64%) will receive no etiological diagnosis.^3^, ^7^, ^8^, ^9^ Recurrent macroscopic haematuria in patients with a previous standard investigation using CTU and cystoscopy negative for UBC or UTUC may prompt further investigations, although recommendations are still unclear.^7^ It is yet to be determined which might be the most appropriate time gap between a primary standard haematuria investigation negative for UBC or UTUC and a subsequent repeated investigation in the case of recurrent macroscopic haematuria. Up to 41% of patients with macroscopic haematuria and previous negative findings will have macroscopic haematuria again; however, the diagnostic yield of a repeated investigation may vary.^7^, ^9^

There are some non‐negligible disadvantages with repeated standard investigations for macroscopic haematuria as cystoscopy, which is invasive procedure with patient discomfort and other complications such as infection and voiding problems. Repeated standard investigations for macroscopic haematuria with cystoscopy has significant limitations that can not be ignored. Cystoscopy is invasive procedure with patient discomfort and other complications such as infection and voiding problems.^10^ Moreover, exposing patients to additional radiation with a repeated CTU is associated with risks of secondary cancers and contrast nephropathy.^11^, ^12^ Furthermore, the financial burden on healthcare facilities and the potential harm caused by excessive investigation of haematuria should not be underestimated.^13^

Thus, the objective of this study was to perform a descriptive analysis of a series of patients with recurrent macroscopic haematuria after a standard primary evaluation that was negative for UBC and UTUC, to explore if there is a reasonable time gap between a primary and a secondary complete evaluation in case of recurrent macroscopic haematuria, and to identify potential factors associated with recurrent macroscopic haematuria.

## 2 MATERIAL AND METHODS

### 2.1 Study subjects

The medical records of all patients evaluated for macroscopic haematuria in the NU Hospital Group (catchment area of 290 000 inhabitants), Uddevalla, Sweden, between 1 January 2015 and 31 December 2017 were retrospectively reviewed. Inclusion criteria were macroscopic haematuria in patients aged 50 years or older who underwent a primary standardized investigation with both cystoscopy and CTU, negative for both UBC and UTUC. Patients with incomplete investigation or diagnosed with either UBC or UTUC at the primary investigation were excluded. A 50‐year limit was chosen based on its similarity to the current SCP criteria and to establish a decision‐making framework that is relevant to the current SCP. Every episode of macroscopic haematuria was recorded, and the timely sequence from the primary investigation and onwards was registered, as was also every diagnosis of urological malignancy. Data were collected from medical records for analysis in March 2023, and patients in the study cohort who had been diagnosed with urological malignancy (UBC, UTUC, RCC or prostate cancer [PC]) either with or without macroscopic haematuria at the end of follow‐up period were identified.

The primary standard investigation included a CTU and a flexible cystoscopy performed by an experienced urologist or a supervised trainee. Urinary cytology was not used routinely, whereas prostate‐specific antigen (PSA) was registered in almost all male patients. At the time of the primary macroscopic haematuria, patients' characteristics and conditions that might potentially contribute to macroscopic haematuria such as presence of indwelling catheter, use of antithrombotic medication (oral anticoagulant, oral antiplatelet or dual medication), presence of urinary tract infection verified with urine culture (UC‐UTI) or history of previous pelvic radiotherapy were recorded. The probable cause of the macroscopic haematuria found during the primary investigation was noted. A normal CTU was considered to be free of pathological findings, whereas all stones in the urinary tracts and all prominent cysts in the kidneys considered to be responsible for macroscopic haematuria were registered. Similarly, a normal cystoscopy was free of pathological findings, whereas all bladder stones, all cases of cystitis and all prominent benign prostate hyperplasia (BPH) considered to be responsible for macroscopic haematuria were recorded.

In the case of recurrent macroscopic haematuria within 1 year, a further investigation was only performed occasionally in some patients. After 1 year, all patients with recurrent macroscopic haematuria had a further investigation using cystoscopy and/or CTU, which was repeated in case of further episodes of macroscopic haematuria. In patients diagnosed with a UBC at the repeated investigation, tumour characteristics including the number of tumours and size, tumour grade according to WHO 1999 classification^14^ and clinical stage according to TNM, 8th Edition^15^ were recorded. A similar approach was conducted regarding newly detected RCC, UTUC and PC. There were no reliable data about smoking. Similarly, data regarding recurrent macroscopic haematuria not reported by the patient nor referred for further examination to our institution were not available.

### 2.2 Statistics

Descriptive statistics were applied for patients' characteristics and outcomes from haematuria investigations. Continuous data were presented as medians with inter‐quartile ranges (IQR). Differences between groups were compared using chi‐squared (Chi2) test for categorical variables and the Mann–Whitney *U* test for continuous variables. Patients were stratified into two groups: either with or without recurrent macroscopic haematuria. The time intervals between primary and repeated investigations were also documented. Follow‐up time started at the primary evaluation and ended at death, detection of a urological malignancy or at the end of observation time on 31 January 2023.

A Poisson regression analysis was used to investigate the association between recurrent macroscopic haematuria and gender, indwelling catheter, antithrombotic medication, having normal primary investigation in cystoscopy or CTU, previous pelvic radiotherapy or UC‐UTI. The analysis was performed with the number of recurrent macroscopic haematuria episodes as the dependent variable and adjusted for follow‐up time. Because of few cancer cases during the follow‐up period, time‐to‐event analysis could not be performed. *P*‐values of <0.05 were considered statistically significant. Statistical analysis was performed using SPSS version 29 (IBM Corp., Armonk, NY, USA).

## 3 RESULTS

**FIGURE 1:**
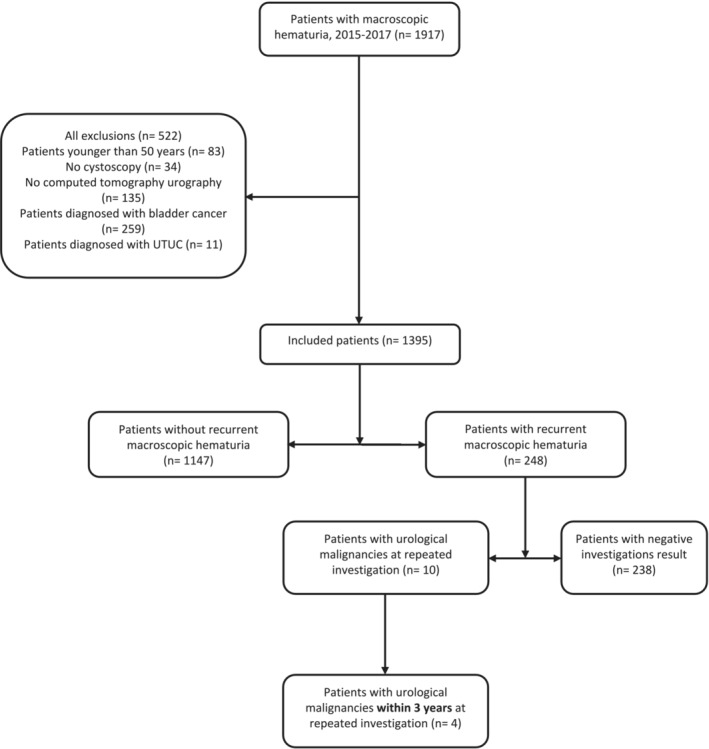
Flow diagram showing inclusion of patients in the study for recurrent macroscopic haematuria.

Of a total of 1917 patients with macroscopic haematuria, 270 patients (14%) were excluded due to a primary investigation positive for UBC or UTUC, of whom 259 patients were diagnosed with UBC and 11 patients with UTUC. Furthermore, patients younger than 50 years (*n* = 83), those who had not undergone cystoscopy (*n* = 34) and patients who had not undergone CTU (*n* = 135) at the primary investigation were excluded (Figure 1). Eligible individuals were 1395 of whom 298 individuals (21%) died during the observation period after a median of 29 (IQR 12–48) months. Survivors had a median follow‐up time of 74 (IQR 65–84) months.

The median age at the primary investigation was 71 (IQR 62–78) years, and 848 patients (61%) were male. At the primary investigation, 475 individuals (34%) had antithrombotic medication, 121 (9%) had an indwelling catheter, 298 (21%) had UC‐UTI and 36 (3%) had previously received pelvic radiotherapy (Table 1). Twenty patients (1.4%) had newly detected RCC at the primary investigation. Three of these 20 patients, all radically treated with surgery and without tumour recurrence, experienced recurrent macroscopic haematuria (due to BPH in two patients and an indwelling catheter‐related issue in the last one). Similarly, nine patients (0.6%) had newly detected PC at the primary investigation, of whom two patients experienced recurrent macroscopic haematuria because of acute cystitis.

**TABLE 1:** Descriptive parameters of all patients investigated for primary macroscopic haematuria by cystoscopy and CTU between 2015 and 2017, stratified into two groups regarding having recurrent macroscopic haematuria or not.

Variable name		Patients without recurrent macroscopic haematuria	Patients with recurrent macroscopic haematuria	All	*p*‐value
No. patients	(% of the row)	1147 (82)	248 (18)	1395	
Gender, *n* (%)	Male	673 (59)	175 (71)	848 (61)	<0.001
Age (years)	Median, (IQR)	70 (62–79)	72 (65–78)	71 (62–78)	0.352
Indwelling catheter	(yes)	97 (9)	24 (10)	121 (9)	0.536
Urine culture verified UTI	(yes)	252 (22)	46 (19)	298 (21)	0.233
Antithrombotic medication	(yes)	375 (33)	100 (40)	475 (34)	0.022
Antithrombotic medication	Antiplatelet	179 (48)	32 (32)	211 (44)	0.009
	Anticoagulant	167 (44)	54 (54)	221 (47)	
	Dual medication	29 (8)	14 (14)	43 (9)	
Previous pelvic radiotherapy	(yes)	26 (2)	10 (4)	36 (3)	0.112
Findings at the primary CTU	Normal	692 (60)	115 (46)	807 (58)	<0.001
	Pathological	455 (40)	133 (54)	588 (42)	
Findings at the primary cystoscopy	Normal	569 (50)	95 (38)	664 (48)	0.001
	Pathological	578 (50)	153 (62)	731 (52)	
Urological malignancies at repeated evaluation for macroscopic haematuria		0 (0)	10 (4)	10 (1)	

*Note*: Figures represent the number of patients (% of the column) if not otherwise indicated.

Abbreviations: BPH, benign prostate hyperplasia; CTU, computed tomography urography; IQR, interquartile range; UBC, urinary bladder cancer; UTI, urinary tract infection; UTUC, upper tract urothelial cancer.

Out of the study cohort, 248 patients (18%) presented with recurrent macroscopic haematuria at a median time of 6.2 (IQR 5.3–7.0) years. At the second evaluation, 185 patients (75%) underwent both cystoscopy and CTU, 57 (23%) had only cystoscopy and six patients (2%) underwent only CTU. Out of these 63 patients without complete secondary evaluation, 12 patients (19%) reported a subsequent episode of macroscopic haematuria of whom 11 patients later had complete third evaluation for recurrent macroscopic haematuria. Fifty‐nine patients (4.2% of the entire cohort) underwent a third investigation (median 4.4 years from the first episode of macroscopic haematuria), 12 patients (1%) had a fourth investigation (after median 4.8 years) and one patient had undergone five investigations. The most common findings at the second investigation were haemorrhagic cystitis (31%) at cystoscopy and benign renal cysts (15%) at CTU. Of the 248 patients that presented with recurrent haematuria, 148 (11% of the entire cohort and 60% of the group with recurrent macroscopic haematuria) presented within 3 years.

### 3.1 Patients diagnosed with urological malignancies at repeated investigation

During the entire follow‐up period, six patients were diagnosed with UBC at the repeated investigation, of whom 83% (5 of 6) were male. All tumours were solitary, and the median size was 1.5 cm. The median time between the first investigation and the time of UBC detection was 2.8 (IQR 1.7–3.7) years (Table 2). Three of these six patients were diagnosed with UBC within 3 years, of whom two had pTaG1, and the third had pT2G3. The latter patient's CTU had significant artefacts because of bilateral hip replacement that made the assessment difficult. Moreover, because of previous radiotherapy for PC, cystoscopy showed abundant radiation cystitis, whereas urinary cytology was lacking, indicating that the primary investigation of this patient might not have been optimal. Furthermore, one male patient had suspected UTUC 6.1 years after the initial investigation, although without pathological verification as the patient refused further diagnostic or therapeutic procedures. In addition, one male patient was diagnosed with a 5‐cm RCC (pT1bN0M0) 4.4 years after the primary investigation, and two patients were diagnosed with PC (Gleason 3 + 3, T1cN0M0 and Gleason 4 + 5, T4N0M0) 2.2 and 4.6 years, respectively, after the primary investigation. No patients were diagnosed with UBC, UTUC or RCC outside the investigations for recurrent haematuria, and 18 patients were diagnosed with PC on the basis of elevated PSA levels.

**TABLE 2:** Patients and tumour characteristics in case of urological malignancies detection at the repeated investigations for recurrent macroscopic haematuria within 6.2 years of the first investigation.

Patient number	1	2	3	4	5	6	7	8	9	10
Gender	Male	Male	Female	Male	Male	Male	Male	Male	Male	Male
Age (years)	74	69	79	66	70	78	53	72	56	80
Cancer type	UBC	UBC	UBC	PC	PC	RCC	UBC	UBC	UBC	UTUC
Number of tumours	1	1	1	NA	NA	1	1	1	1	1
Tumour size (cm)	2	2	0.5	NA	NA	5	1	2	0.5	5
Tumour stage	TaG1	T2G3	TaG1	T1c, GL3+3	T4, GL4+5	T1b	TaG2	T2G3	TaG1	NA
Time from the first investigation (year)	1.6	1.8	2.3	2.2	3.2	3.2	3.2	3.3	4.8	6.1
Order of the further investigation	2nd	2nd	2nd	2nd	4th	2nd	2nd	2nd	3rd	2nd

Abbreviations: GL, Gleason score; PC, prostate cancer; RCC, renal cell carcinoma; UBC, urinary bladder cancer; UTUC, upper tract urothelial cancer.

### 3.2 Analysis of patients with and without recurrent macroscopic haematuria

Patients were stratified into two groups depending on the presence or absence of macroscopic haematuria. There were more males (71% compared with 59%, *p* < 0.001) and more patients taking antithrombotic medication in the group with recurrent macroscopic haematuria (40% compared with 33%, *p* = 0.022). Similarly, in the group with recurrent macroscopic haematuria, there was a larger proportion of patients with pathological findings at the primary investigation at both cystoscopy (62% compared with 50%, *p* = 0.004) and CTU (54% compared with 40%, *p* = 0.003).

After adjusting for follow‐up time and considering the number of recurrent macroscopic haematuria episodes, Poisson regression multivariate analysis showed that presence of indwelling catheter (rate ratio [RR] 3.45, 95% CI 2.25–5.29, *p* < 0.001), use of antithrombotic medication (RR 3.63, 95% CI 2.84–4.64, *p* < 0.001), presence of pathological finding at CTU (RR 3.34, 95% CI 2.59–4.32, *p* < 0.001) or at cystoscopy (RR 1.49, 95% CI 1.12–1.98, *p* = 0.006) and history of previous pelvic radiotherapy (RR 6.59, 95% CI 3.71–11.71, *p* < 0.001) were statistically significant independent risk factors for having recurrent macroscopic haematuria (Table 3). Conversely, the presence of urine culture verified UTI (RR 0.23, 95% CI 0.16–0.34, *p* < 0.001) was inversely associated with recurrence of haematuria.

**TABLE 3:** Poisson regression analysis performed on all patients with primary macroscopic haematuria in NU Hospital Group with adjustment for follow up time and with (the number of recurrent haematuria episodes during the follow up period) as a dependent variable.

Variable name	RR, Univariate (95% CI)	*p*‐value	RR, Multivariate (95% CI)	*p*‐value
Gender (Male)	1.42 (1.11–1.81)	0.006	0.34 (0.25–0.48)	<0.001
Indwelling catheter	1.94 (1.34–2.83)	<0.001	3.45 (2.25–5.29)	<0.001
Antithrombotic medication	3.35 (2.68–4.19)	<0.001	3.63 (2.84–4.64)	<0.001
Pathological findings at the primary CTU	2.54 (2.04–3.16)	<0.001	3.34 (2.59–4.32)	<0.001
Pathological findings at the primary cystoscopy	1.84 (1.46–2.30)	<0.001	1.49 (1.12–1.98)	0.006
Previous pelvic radiotherapy	2.74 (1.63–4.59)	<0.001	6.59 (3.71–11.71)	<0.001
Urine culture verified UTI	0.44 (0.33–0.60)	<0.001	0.23 (0.16–0.34)	<0.001

Abbreviations: CI, confidence interval; CTU, computed tomography urography; RR, rate ratio.

## 4 DISCUSSION

Repeated episodes of macroscopic haematuria after an initial negative investigation are common clinical settings, but there is no consensus for when a repeated investigation should be done. In the present study, and after a primary standard evaluation negative for UBC and UTUC, we found urological malignancies in 10 patients (4%) presenting with recurrent macroscopic haematuria, but only four individuals (2.7%) within 3 years of the primary investigation. These findings are in line with the findings by Mullen et al.^16^ who presented a low yield of repeated CTU for diagnosing a malignancy within 3 years for patients with recurrent macroscopic haematuria. High diagnostic accuracy of UBC using only CTU has previously been demonstrated^17^ underscoring the observation by Mullen et al.^16^

Our results are also supported by the analysis of Sells et al. studying 26 patients with recurrent macroscopic haematuria with at least 2‐year follow‐up time after the initial investigation.^18^ They found that one patient had UTUC already visible at the primary CTU.^18^ Therefore, a repeated investigation with cystoscopy and CTU was proposed to be only warranted in selective patients. In the present study and within 3 years from the primary investigation negative for UBC and UTUC, only four urological malignancies were reported. One of these had a primary suboptimal investigation because of serious problems in both CTU and cystoscopy regarding the interpretation of findings and harbouring muscle invasive UBC at repeated investigation. As the other tumours were low malignant including two TaG1 UBC and one small PC Gleason (3 + 3), it may be reasonable to extend the time gap between the first and the second investigation to 3 years. However, our results also indicate that, before such a decision to postpone a second investigation is taken, a primary investigation of good quality is mandatory.

In contrast to our results, some studies had found a higher number of urological malignancies on repeated investigation.^7^, ^9^, ^19^ Rasmussen et al.^19^ found urological malignancies in seven of 38 patients (18%) who had recurrent macroscopic haematuria after primary investigation with cystoscopy and conventional urography. However, the study is nearly 35 years old and applied urography and not CTU with presumed higher sensitivity to detect malignancies. Moreover, five of these seven patients (71%) presented with non‐muscle invasive bladder cancer, of whom only two were detected within the first 3 years. A similar proportion of malignancies at repeated investigation was observed by Mishriki et al.,^7^ reporting that 12 of 69 patients (17%) with recurrent macroscopic haematuria had urological malignancies (PC *n* = 4, UBC *n* = 5, RCC *n* = 2 and UTUC in one patient) at the repeated investigation. However, the earliest diagnosis was made after 3.8 years. The high yield of UBC in this study may be explained by that 83% of their cohort were men (compared with 61% in our cohort), considering that men have a higher risk of UBC than women.

In an earlier study by Mishriki et al.,^9^ four of 41 patients (9.8%) with recurrent macroscopic haematuria had a urological malignancy. Although CTU was not available as the standard investigation in that study, all included patients in our study had undergone a primary complete investigation with both cystoscopy and CTU. Compared with the current study, all these three studies may have limited statistical power because of the small number of participants. Additionally, the routine investigations in these studies were not always complete using both cystoscopy and CTU.

In the present study, patients with indwelling catheter, antithrombotic medication, pathological findings at cystoscopy and CTU, history of previous pelvic radiotherapy or absence of UC‐UTI seem to be associated with significant increased risk for recurrent macroscopic haematuria. This underscores the importance of conducting a thorough and prioritized investigation in patients with these risk factors to ensure that no malignancies are overlooked when managing primary macroscopic haematuria. Moreover, risk factors, such as urinary stones or BPH, should be actively treated in order to avoid recurrent macroscopic haematuria. Pelvic radiotherapy (RT) plays an important role in the management of many cancers in the pelvis with both early and late morbidity.^20^ Moreover, radiation cystitis is a real challenge, and such lesions might delay the diagnosis of UBC, as was probably the case in one of our patients. Therefore, in patients with a history of pelvic radiotherapy and current macroscopic haematuria, urinary cytology and biopsy should generously be taken to exclude malignancy.

Within 3‐year follow‐up of patients with recurrent macroscopic haematuria, three patients had low malignant urological cancer. Thus, a routinely performed repeated standard evaluation may be unnecessary within this timeframe, which can lower risks and discomfort associated with invasive procedures and minimize the potential harm from radiation exposure and radiation‐induced secondary malignancy^21^ and conserve healthcare resources. Since the diagnostic yield appears to be low, a more selective approach regarding repeated investigations with less resource‐intensive and less invasive methods such as cancer markers or cytology of voided urine might be considered.^22^ Furthermore, treatment of underlying causes of recurrent haematuria might be prioritized in order to minimize recurrences and the need for repeated investigation. Antibiotics for UTIs, surgery or medical treatment with alpha reductase inhibitors for BPH or treating stones can be considered.^23^ However, in patients with recurrent macroscopic haematuria with previous inconclusive cystoscopy or CTU, we still strongly recommend a further investigation.

Nonetheless, postponing the investigation for a period of 3 years could pose challenges and potentially result in a delayed diagnosis of urological malignancies. One of our patients had cT2 UBC within a relatively short interval of 1.8 years, and further delaying the diagnosis by 1.2 years could have potentially caused significant harm. However, it is important to note that in this specific case, substantial artefacts in the CTU due to bilateral hip replacement and the presence of advanced radiation‐induced cystitis due to prior radiotherapy for PC may have made the assessment inconclusive from the outset. Such factors and in case of difficulties to ensure a good quality primary investigation in a patient with recurrent macroscopic haematuria a new investigation should be performed on wide indications.

To the best of our knowledge, this is the first study to examine the natural course of recurrent macroscopic haematuria in a large cohort with previously negative results for UBC and UTUC at a primary standard investigation. Based on our data, it appears reasonable to avoid a repeated full investigation for all patients with recurrent macroscopic haematuria within 3 years of the primary investigation, which is also consistent with a previous study.^16^ This new period was selected to balance the need to avoid missing other possible urological malignancies that may develop independently of the initial episode of macroscopic haematuria, while still being short enough to detect any pathological entities in a timely manner.

Strengths of this study are the substantial sample size and the originality of its research concept, particularly in an area with limited existing evidence or guidance. Furthermore, the study included all patients with macroscopic haematuria between 2015 and 2017 within a delimited geographical region, benefiting from a well‐established primary care system that reduces the likelihood of missing information regarding episodes of macroscopic haematuria. Moreover, the study has a median follow‐up period of 6.2 years, which provides a considerable time period to observe the natural course of recurrent macroscopic haematuria and evaluate the risk of malignancy detection during the follow‐up time. Nevertheless, this study lacks essential information about smoking habits of the participants. Moreover, lack of routine urinary cytology and not having a standard re‐investigation with both CTU and cystoscopy for all patients are limitations of our study, although in case of further episodes of macroscopic haematuria, a complete investigation was mostly done. Furthermore, even if some episodes of recurrent haematuria were potentially missed, the fact that no cases of UBC or UTUC were identified outside the haematuria investigations suggests that this is a minor limitation. Additionally, the low incidence of malignancies detected in the recurrent macroscopic haematuria cohort posed difficulties in conducting time‐to‐event statistical analysis that highlights the necessity for larger, multicentre and prospective studies to validate our findings.

## 5 CONCLUSIONS

In the case of recurrent macroscopic haematuria within 3 years, there was a low risk for serious urological malignancies in patients with a primary standard investigation negative for UBC and UTUC. As a result, waiting 3 years before conducting another complete standard investigation in cases of recurrent macroscopic haematuria appears to be appropriate. However, unexplained, recurrent or persistent macroscopic haematuria with inconclusive previous cystoscopy or poor quality CTU should still demand further evaluation.

## AUTHOR CONTRIBUTIONS

**Suleiman Abuhasanein:** Conceptualization; methodology; formal analysis; data curation; writing—original draft preparation. **Vanessa Chaves:** Data review; writing—reviewing and editing. **Ali Moustafa Mohsen:** Data review; writing—reviewing and editing. **Jasmine Al‐Haddad:** Data review; writing—reviewing and editing. **Merete Sunila:** Data review; writing—reviewing and editing. **Viveka Ströck:** Writing—reviewing and editing. **Tomas Jerlström:** Writing—reviewing and editing. **Fredrik Liedberg:** Writing—reviewing and editing. **Jesper Swärd:** Writing—reviewing and editing. **Truls Gårdmark:** Writing—reviewing and editing. **Staffan Jahnson:** Supervision; writing—reviewing and editing. **Henrik Kjölhede:** Supervision; writing—reviewing and editing.

## CONFLICT OF INTEREST STATEMENT

The authors have no conflict of interest to declare.
